# The First Survival Score for Patients Treated with Whole-Brain Radiotherapy Plus Simultaneous Integrated Boost for Brain Metastases

**DOI:** 10.3390/biology12040585

**Published:** 2023-04-12

**Authors:** Dirk Rades, Leonie Johannwerner, Elisa M. Werner, Florian Cremers, Nathan Y. Yu

**Affiliations:** 1 Department of Radiation Oncology, University of Lubeck, 23562 Lubeck, Germany; leonie.johannwerner@web.de (L.J.); elisamarie.werner@uksh.de (E.M.W.); florian.cremers@uksh.de (F.C.); 2 Department of Radiation Oncology, Mayo Clinic, Phoenix, AZ 85054, USA; yu.nathan@mayo.edu

**Keywords:** cerebral metastases, whole-brain radiotherapy, simultaneous integrated boost, survival, predictive model

## Abstract

**Simple Summary:**

A model was generated to judge survival after whole-brain radiotherapy plus simultaneous boost (WBRT+SIB) for cerebral metastases. Initially, three models, each including three prognostic groups, were created and compared for accuracy in prognosticating death ≤ 6 and survival ≥ 6 months using the corresponding positive predictive values (PPVs). PPVs to predict death ≤ 6 and survival ≥ 6 months were 85% and 57% for Model 1 (considering performance status = KPS and the number of lesions), 83% and 75% for Model 2 (KPS, lesions, age), and 86% and 78% for Model 3 (KPS, lesions, age, extra-cerebral metastases), respectively. The three models were accurate in predicting death ≤ 6 months and Models 2 and 3 were more precise than Model 1 in predicting survival ≥6 months. Model 2 appears preferable for many patients. Patients with poor survival prognoses may not benefit from adding SIB.

**Abstract:**

A modern approach for brain metastases includes whole-brain radiotherapy plus simultaneous boost (WBRT+SIB). We developed a survival score in 128 patients treated with WBRT+SIB. Three models, each including three prognostic groups, were created. Positive predictive values (PPVs) for death ≤6 and survival ≥6 months were calculated. On multivariate analyses, performance score (KPS) and the number of brain metastases were significantly associated with survival. On univariate analyses, age showed a strong trend, and extra-cerebral cranial metastases a trend. In Model 1 (KPS, number of lesions), compared groups had 6-month survival rates of 15%, 38% and 57%. In Model 2 (KPS, lesions, age), rates were 17%, 33% and 75%, and in Model 3 (KPS, lesions, age, extra-cerebral metastases), 14%, 34% and 78%. PPVs for death ≤6 and survival ≥6 months were 85% and 57% (Model 1), 83% and 75% (Model 2), and 86% and 78% (Model 3). Thus, all models were accurate in predicting death ≤ 6 months; poor-prognosis patients may not benefit from SIB. Models 2 and 3 were superior in predicting survival ≥ 6 months. Given that Model 3 requires more data (including extensive staging), Model 2 is considered favorable for many patients. If extra-cerebral metastases are already known or extensive staging has been performed, Model 3 can also be used.

## 1. Introduction

Cerebral metastases affect a significant number of patients following their diagnosis of cancer [1,2]. The majority of these patients are treated with radiation therapy, either alone or in combination with other treatment modalities. Radiotherapy techniques for brain metastases include whole-brain radiotherapy (WBRT) and local radiation approaches, namely stereotactic radiosurgery (one fraction with a high dose of 15–24 Gy) and fractionated stereotactic radiotherapy (generally 27–30 Gy given in 3 to 5 fractions) [1,2]. In previous studies, these techniques were combined as a sequential approach (WBRT plus a stereotactic boost) for very few cerebral metastases [3,4]. In those studies, the addition of a stereotactic boost to WBRT improved intra-cerebral control. Another approach combines WBRT with an integrated boost (SIB). Modern radiation therapy techniques, such as volumetric modulated arc therapy (VMAT), can generate steep dose gradients between the outer margin of the metastases and the surrounding normal tissues [5]. Thus, VMAT allows the delivery of lower doses to the whole brain and increased doses to the metastatic lesions in one treatment plan during the same radiation session [5]. This approach generally reduces treatment time and dose to normal organs [5,6,7,8,9]. Earlier feasibility studies using helical tomotherapy produced promising results [10,11] that were confirmed in more recent studies using intensity-modulated radiation therapy (IMRT) [12,13,14]. In a study from 2022, even the combination of WBRT+SIB and the tyrosine kinase inhibitor apatinib appeared safe [15]. Moreover, in several studies, WBRT+SIB was similarly effective as stereotactic radiotherapy, WBRT, or a combination of both with respect to local control or intra-cerebral control [6,16,17,18,19]. Some studies even suggested that WBRT+SIB resulted in improved outcomes compared to WBRT with or without stereotactic radiosurgery [7,20,21,22]. Based on these results, interest has increased in using WBRT+SIB, including the combination with hippocampus-sparing techniques to reduce the risk of neurocognitive decline [18,19,20,21,22,23,24,25]. However, not all patients considered candidates for WBRT may benefit from the addition of a SIB, especially patients with poor prognoses. To identify patients who may not benefit from an additional SIB, we developed a survival score specifically for patients who are considered for WBRT+SIB.

## 2. Patients and Methods

**Figure 1:**
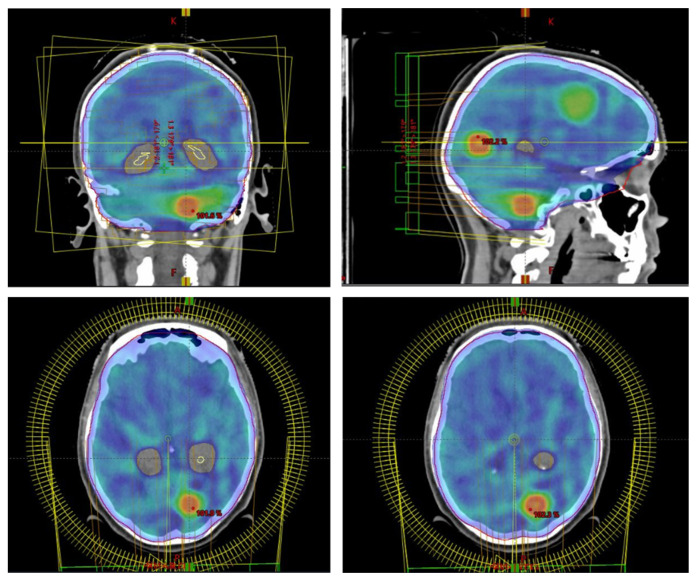
Example of a treatment plan of whole-brain radiotherapy plus a simultaneous integrated boost to the metastatic lesions using a hippocampus-sparing technique (Department of Radiation Oncology, University of Lübeck, Germany).

One-hundred-and-twenty-eight patients with newly diagnosed metastases to the brain, who were not considered candidates for neurosurgical resection or stereotactic radiotherapy by a multidisciplinary tumor board, were analyzed. This retrospective study received approval from the Ethics Committee of the University of Lübeck, Lübeck, Germany (22-059). All 128 patients were treated with WBRT plus a SIB to metastatic sites between 2014 and 2021 using VMAT (an example of a treatment plan given in Figure 1). Eighty-seven patients had 1–5 brain metastases, 25 patients had 6–10 lesions, and 16 patients had >10 lesions, respectively. The radiation regimens were either 14 × 2.5 Gy of WBRT plus a SIB of 0.5 Gy per fraction, resulting in a total dose of 42 Gy to the metastatic sites (49 patients), or 18 × 2.0 Gy of WBRT plus a SIB of 0.5 Gy per fraction resulting in a total dose of 45 Gy to the brain lesions (79 patients). The corresponding biologically effective doses using an alpha/beta ratio of 12 Gy for control of brain metastases were 42.3 Gy_12_ to the entire brain and 52.5 Gy_12_ to the metastases, respectively, for 14 × 2.5 Gy of WBRT plus a SIB of 0.5 Gy per fraction, and 42.0 Gy_12_ and 54.4 Gy_12_, respectively, for 18 × 2.0 Gy of WBRT plus a SIB of 0.5 Gy per fraction.

Ten factors (Table 1) were investigated for associations with survival, including the year of treatment (2014–2018 vs. 2019–2021), time between diagnosis of malignancy and the first fraction of radiation therapy (0–1 vs. ≥2 months), radiotherapy regimen (14 × 2.5 Gy of WBRT plus SIB vs. 18 × 2.0 Gy of WBRT plus SIB), systemic treatment with 6 months before radiotherapy (no vs. yes), age at radiotherapy (≤64 vs. ≥65 years, median = 64 years), gender (female vs. male), Karnofsky performance score (KPS ≤80 vs. 90–100), primary tumor entity (breast cancer vs. non-small cell lung cancer vs. small-cell lung cancer vs. less radiosensitive tumors including kidney cancer and melanoma vs. other types), number of brain lesions (1–3 vs. ≥4, median = 3), and the existence of extra-cerebral metastases (no vs. yes). The year of treatment was included as a parameter since the use of targeted therapies has increased in recent years, particularly for patients with non-small cell lung cancer [15]. Often, these targeted therapies lead to improved survival.

Patients were followed until they died or for at least 6 months after the end of their radiotherapy course. For univariate analyses, we used the Kaplan-Meier method supplemented by the log-rank test (BlueSky Statistics 10 GA, Chicago, IL, USA). Characteristics found significant in univariate analyses (*p* < 0.05) or indicating a strong trend (*p* < 0.07) or a trend (*p* < 0.14) were included in multivariate analyses (Cox proportional hazards model). Those factors that achieved significance in the Cox proportional hazards model were considered independent predictors of survival. Therefore, we felt that it was appropriate to perform both univariate and multivariate analyses. To provide the most precise survival score, three different models were calculated. Model 1 included only factors being significant on both univariate and multivariate analyses (=independent factors), Model 2 included factors that indicated at least a strong trend in univariate analyses, and Model 3 included factors indicating a trend. For each factor included in one of the three models, the 6-month survival rates (in %) were divided by 10. Scoring points obtained from this procedure (factor scores) were added individually for each patient (patient scores). Higher patient scores represented better 6-month survival. Thus, the three models are not mathematical models but were created from real patient data, namely from the patients’ 6-month survival rates. Therefore, we can estimate the probability of dying within 6 months following WBRT+SIB and living 6 or more months following WBRT+SIB.

Based on the corresponding 6-month survival rates, three groups (poor, intermediate, and favorable prognosis) were created. The models were compared for diagnostic accuracy by calculating positive predictive values (PPVs) to correctly prognosticate death during the 6 months following radiotherapy (least favorable prognostic groups) and survival for 6 or more months (most favorable prognostic groups).

PPVs to correctly predict death within 6 months were calculated as follows:

*PPV* = [*patients dying*/(*patients dying* + *patients not dying*)] × 100 (1)

PPVs to correctly predict survival for at least 6 months were calculated as follows:

*PPV* = [*patients surviving*/(*patients surviving* + *patients not surviving*)] × 100 (2)

## 3. Results

On univariate analyses, KPS of 90–100 (*p* < 0.001) and only 1–3 brain lesions (*p* = 0.006) were significantly associated with better survival. Age ≤ 64 years (*p* = 0.061) showed a strong trend, and no metastases outside the brain (*p* = 0.128) showed a trend. The results of the complete univariate analyses, including the survival rates at 3, 6, and 12 months are summarized in Table 2. On multivariate analyses, KPS [hazard ratio (HR) 0.40, 95% confidence interval (CI) 0.27–0.61, *p* < 0.001) and the number of lesions (HR 1.81, 95% CI 1.23–2.65, *p* = 0.002) remained significant, whereas age (HR 1.12, 95% CI 0.76–1.66, *p* = 0.57) and extra-cerebral metastases (HR: 1.19, 95% CI: 0.79–1.78, *p* = 0.40) did not reach significance. The scoring points for each factor are shown in Table 3. These factor scores were valid for each model. Since the models included different numbers of factors, patient scores (obtained after the addition of the factor scores) were also different. Patient scores ranged between 5 and 9 points in Model 1, between 8 and 13 points in Model 2, and between 11 and 17 points in Model 3.

**Figure 2:**
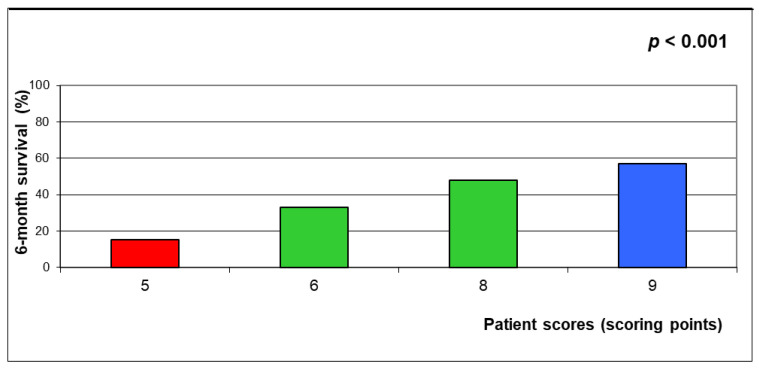
Model 1 (including KPS and number of lesions): Six-month survival rates of patient scores ranging between 5 and 9 points.

**Figure 3:**
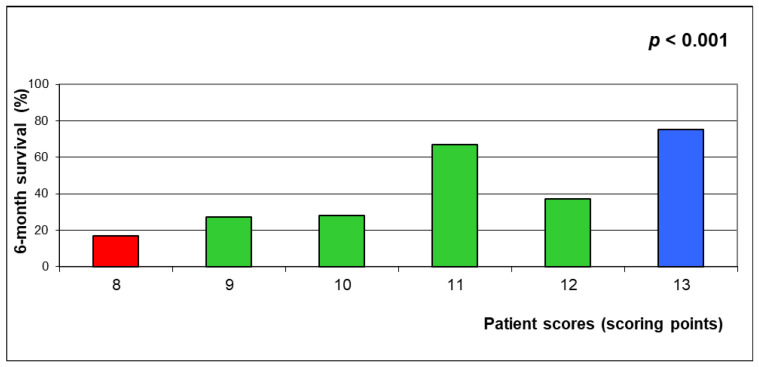
Model 2 (including KPS, number of lesions, and age): Six-month survival rates of patient scores ranging between 8 and 13 points.

**Figure 4:**
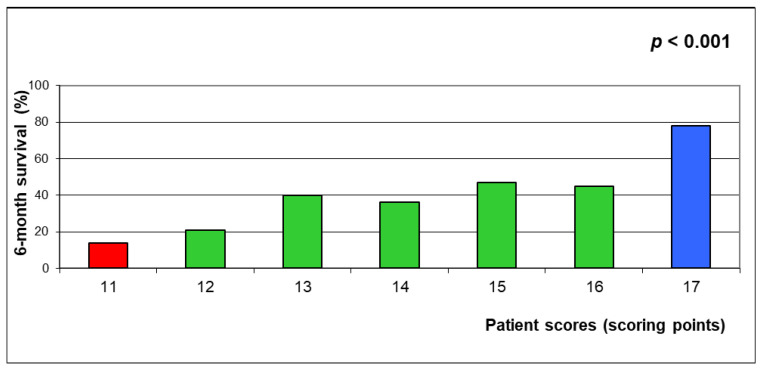
Model 3 (including KPS, number of lesions, age, and extra-cerebral metastases): Six-month survival rates of patient scores ranging between 11 and 17 points.

The 6-months survival rates of the patient scores of the three models are shown in Figure 2, Figure 3 and Figure 4.

**Figure 5:**
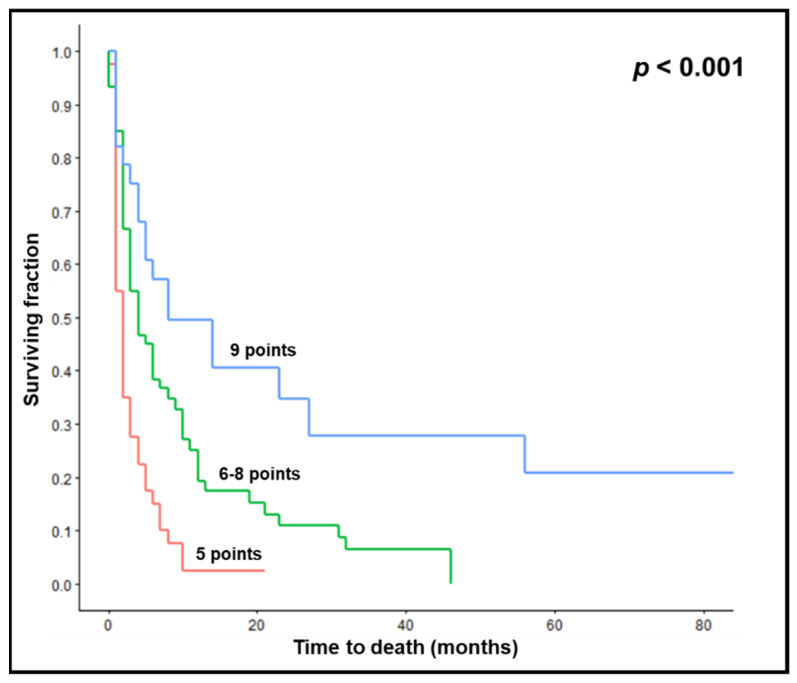
Model 1: Kaplan-Meier curves for the survival of the three groups (5, 6–8 and 9 points).

**Figure 6:**
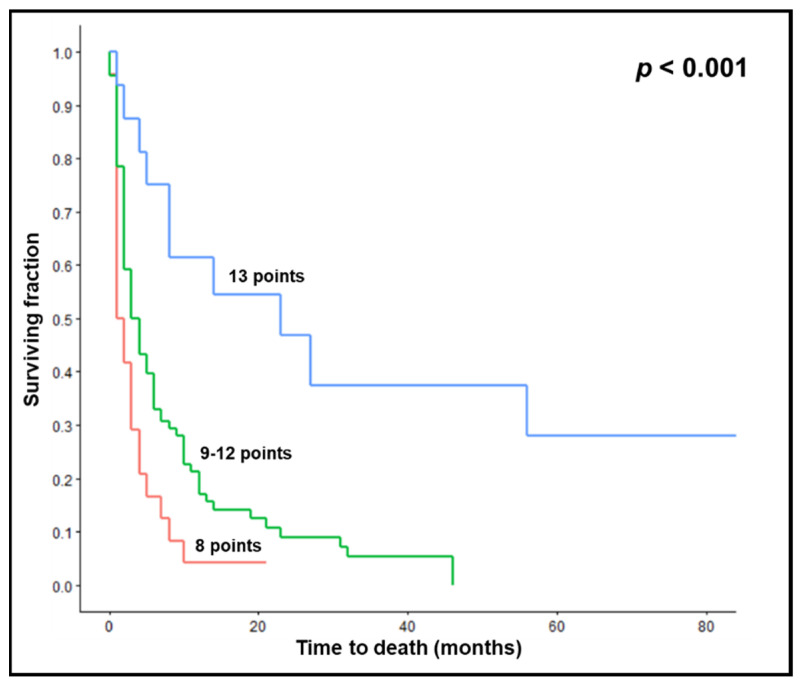
Model 2: Kaplan-Meier curves for survival of the three groups (8, 9–12, and 13 points).

**Figure 7:**
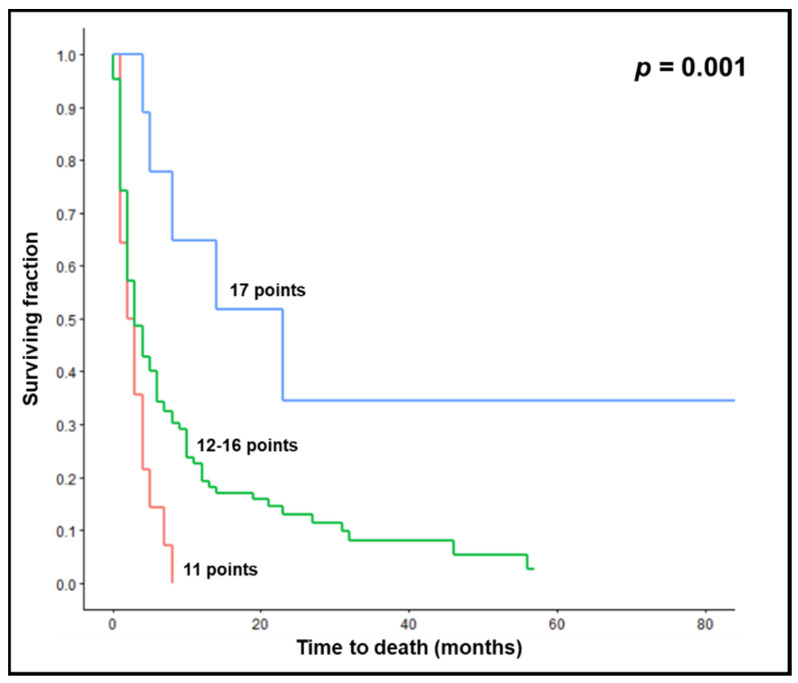
Model 3: Kaplan-Meier curves for survival of the three groups (11, 12–16 and 17 points).

Based on the 6-month survival rates of the patient scores, three groups with different survival outcomes were created for each model. In Model 1 (including KPS and the number of lesions), groups were 5 points, 6–8 points, and 9 points with 6-month survival rates of 15%, 38%, and 57%, respectively (*p* < 0.001, Figure 5). In Model 2 (including KPS, number of lesions, and age), groups were 8 points, 9–12 points, and 13 points with 6-month survival rates of 17%, 33%, and 75%, respectively (*p* < 0.001, Figure 6). In Model 3 (including KPS, number of lesions, age, and extra-cerebral metastases), groups were 11 points, 12–16 points, and 17 points, with 6-month survival rates of 14%, 34%, and 78%, respectively (*p* = 0.001, Figure 7). The PPVs for accurate prediction of death during 6 months and survival for 6 or more months were 85% and 57% for Model 1, 83% and 75% for Model 2, and 86% and 78% for Model 3, respectively.

## 4. Discussion

Different techniques and regimens are available for radiotherapy of brain metastases. A regimen that is increasingly used includes WBRT plus a SIB. Compared to WBRT plus a sequential boost, WBRT+SIB has two advantages, i.e., a reduced treatment time and dose to normal tissues [5,6,7,8,9]. Several studies have shown that WBRT+SIB is feasible. Fifteen years ago, Tomita et al. observed no acute toxicity or late complications (median follow-up = 10 months) in seven patients with 1–4 brain metastases treated with 10 × 3 Gy of WBRT and a SIB of 2.0 Gy per fraction to metastatic locations using helical tomotherapy [10]. In 2011, the results of a phase I trial were reported [11]. Patients (*n* = 48) had 1–3 brain metastases and received 10 × 3 Gy of WBRT plus a SIB with increasing doses ranging between 0.5 and 3.0 Gy per fraction (3 + 3 design). No patient, including those receiving 60 Gy in 10 fractions to the metastatic lesions, experienced dose-limiting neurological toxicity [10]. A similar approach was chosen by Ferro et al. in 2017 [12]. A total of 30 patients were treated with 10 × 3 Gy of WBRT plus a SIB for 1–4 brain metastases using IMRT. SIB doses ranged between 0.5 and 2.0 Gy per fraction. Three patients experienced dose-limiting toxicity, including one grade 3 dermatitis at dose level 2, one grade neurological toxicity at dose level 4, and one intracerebral hemorrhage at dose level 4, respectively [12]. In the retrospective study of Dong et al., 15 × 2.5 Gy of WBRT plus a SIB of 1.0 Gy per fraction was found feasible and safe in 46 patients with 1–7 brain metastases from non-small cell lung cancer [13]. Moreover, in a more recent phase II trial of 50 patients with 1–8 brain metastases receiving 10 × 2.0 Gy of hippocampus-sparing-WBRT plus a SIB of 4.0 Gy per fraction using IMRT or VMAT, only three patients experienced grade 3 toxicities, including nausea, emesis, and necrosis or headache [14]. Moreover, in a recent retrospective multi-center study, the combination of WBRT+SIB and the tyrosine kinase inhibitor apatinib was both feasible and effective [15]. In that study, oral apatinib was started one week before radiation therapy and administered during and for one week following this treatment. Two of 16 patients (12.5%) developed a grade 3 toxicity, namely oral mucositis and hypertension. At three months following treatment, intra-cerebral control was 100%. Median survival and intra-cerebral progression-free survival times were 26 months and 16.5 months, respectively [15].

In addition to demonstrating the feasibility of WBRT+SIB, several studies have shown that this approach leads to local and intra-cerebral control rates at least comparable to other radiation approaches, namely WBRT alone, stereotactic radiotherapy alone, or WBRT plus stereotactic radiotherapy [6,16,17,18,19]. Some data suggests that WBRT+SIB may be superior to WBRT with or without stereotactic radiosurgery [7,20,21,22]. For example, in the most recent study, WBRT+SIB resulted in longer median intra-cerebral progression-free survival than WBRT, followed by stereotactic radiosurgery (91 vs. 5.0 months, *p* = 0.001) [22]. However, despite increasing interest in using WBRT+SIB, particularly with hippocampus-sparing techniques, not all patients may benefit from this approach.

Survival scores can facilitate identifying patients who may or may not benefit from a WBRT+SIB. Several scores were already developed for patients with brain metastases, including tools for specific techniques and approaches [26,27,28,29,30,31,32]. However, no such tool exists for patients receiving WBRT+SIB. We developed a survival score in a cohort of 128 patients treated with WBRT+SIB and compared three models, including different prognostic factors. Model 1 included two factors, namely KPS and the number of lesions. Model 2 was supplemented by age, and Model 3 was supplemented by age and extra-cerebral metastases. These prognostic factors were previously used in different constellations for survival scores that were designed for patients irradiated for brain metastases but not specifically for those patients receiving WBRT+SIB [26,27,28,29,30,31,32]. These findings demonstrate the consistency of the results in our present study. However, our results should be interpreted with caution, given the retrospective nature of this study.

The main purpose of the three models investigated in this study was the prediction of death within 6 months following WBRT+SIB. The second goal was the prediction of survival for at least 6 months. All three models provided high accuracy regarding the prediction of death within 6 months, with PPVs of 85%, 83%, and 86%, respectively. Regarding the second goal, Model 2 and Model 3 were superior to Model 1 (PPVs of 75% and 78%, respectively, compared to 57%). When comparing Models 2 and 3, the differences regarding the accuracy to predict death ≤6 and survival ≥6 months appear marginal. Given that Model 3 requires more data (including extensive staging to properly judge the presence of extra-cerebral metastases) and is only marginally more accurate than Model 2, Model 2 appears favorable for a considerable number of patients. If extra-cerebral metastases have already been diagnosed or extensive staging is already available without a diagnosis of metastases outside the brain, Model 3 can also be used. When using model 2, those patients with 8 points likely did not benefit from the addition of a SIB since the 6-month survival rate was poor (17%), and the median survival time was only 1.5 months. This likely holds true also for many patients with 9–12 points (6-month survival rate of 33% and median survival time of 3.5 months, respectively). Thus, only patients with 13 points appear as good candidates for adding a SIB. When using Model 3, patients with 17 points appear suitable for the addition of a SIB since the 6-month survival rates of the other two groups (11 points and 12–16 points) were only 14% and 34%, respectively.

However, as stated above, if one considers following the treatment suggestions based on this study’s findings, its limitations must be kept in mind, most of all the retrospective nature of the data used to generate the predictive models. Moreover, many patients with up to 3 or 4 cerebral lesions are candidates for stereotactic radiotherapy with stereotactic radiosurgery in one fraction or multi-fraction stereotactic radiotherapy alone rather than for WBRT+SIB [1,2]. In a study from 2007 including 186 patients with 1–3 brain metastases, 18–25 Gy of stereotactic radiosurgery alone resulted in similar survival and better local control (risk ratio = 1.63, *p* < 0.01) than 30–40 Gy (2–3 Gy per fraction) of WBRT alone [33]. To further improve radiation therapy outcomes for a limited number of cerebral metastases, several studies investigated the combination of stereotactic radiosurgery and WBRT and compared this approach to stereotactic radiosurgery alone [34,35,36,37,38,39]. In these studies, the addition of WBRT resulted in better intra-cerebral control but not in better survival. Moreover, in two randomized trials, the addition of WBRT resulted in significantly more neuro-cognitive deficits [38,39]. Therefore, many radiation oncologists prefer stereotactic radiosurgery alone rather than stereotactic radiosurgery in combination with WBRT for 1–3 cerebral metastases. This holds particularly true for patients with a low or intermediate risk of developing new brain metastases outside the irradiated cerebral areas within a short period of time. In 2014, a predictive model including three groups was presented that estimated the risk of new brain metastases within 6 months after stereotactic radiotherapy alone [40]. The risk of new cerebral lesions during 6 months post radiation therapy was 36%, 55%, and 80%, respectively, for patients with 16–17, 18–20 and 21–22 points). If stereotactic radiotherapy is used for very few cerebral metastases, multi-fraction stereotactic radiotherapy, for example, with 3 × 9 Gy or 5 × 6 Gy, is preferred over single-fraction treatment for lesions >2 cm in largest diameter since multi-fraction stereotactic irradiation results in less radiation necrosis than single-fraction radiosurgery [1,41]. In a study with 289 consecutive patients with lesions >2 cm from Italy, the cumulative radiation necrosis rates were 18% after single-fraction radiosurgery and 9% after multi-fraction treatment, respectively [41].

## 5. Conclusions

A new survival score was developed for patients with brain metastases assigned to WBRT+SIB. Three models were created and compared for accuracy. All models accurately predicted death ≤ 6 months with PPVs of 85%, 83%, and 86%, respectively. This can help identify patients who may not benefit from WBRT+SIB. Regarding the prediction of survival for at least 6 months, Model 2 and Model 3 were superior to Model 1, with PPVs of 75% and 78% compared to 57%. Given that Model 3 requires extensive staging to properly judge the existence of metastases outside the brain and is only marginally more accurate than Model 2, Model 2 appears favorable for many patients. If metastases outside the brain are already known and/or extensive staging has already been performed, Model 3 can also be used. Validation of these Models in a prospective cohort of patients is warranted.

## Figures and Tables

**Table 1:** Distribution of the factors analyzed for survival.

Factor	Number of Patients	Proportion (%)
Year of treatment		
2014–2018	72	56
2019–2021	56	44
Time between tumor diagnosis and RT		
0–1 months	70	55
≥2 months	58	45
RT regimen		
14 × 2.5 Gy of WBRT + SIB	49	38
18 × 2.0 Gy of WBRT + SIB	79	62
Pre-RT systemic treatment		
No	79	62
Yes	49	38
Age at RT		
≤64 years	65	51
≥65 years	63	49
Gender		
Female	49	38
Male	79	62
Karnofsky performance score		
≤80	79	62
90–100	49	38
Type of primary tumor		
Breast cancer	8	6
Non-small cell lung cancer	77	60
Small-cell lung cancer	18	14
Less radiosensitive tumors	13	10
Other types	12	9
Number of brain lesions		
1–3	67	52
≥4	61	48
Extra-cerebral metastases		
No	47	37
Yes	81	63

RT: Radiotherapy; WBRT: Whole-Brain Radiotherapy; SIB: Simultaneous Integrated Boost.

**Table 2:** Survival rates of the investigated factors at 3, 6, and 12 months following radiotherapy.

Factor	Survival Rate (%)			*p*-Value
	at 3 Months	at 6 Months	at 12 Months	
Year of treatment				0.34
2014–2018	51	36	23	
2019–2021	50	34	17	
Time between tumor diagnosis and RT				0.56
0–1 months	56	40	23	
≥2 months	45	29	18	
RT regimen				0.55
14 x 2.5 Gy of WBRT + SIB	55	37	22	
18 x 2.0 Gy of WBRT + SIB	48	34	19	
Pre-RT systemic treatment				0.84
No	52	35	23	
Yes	49	35	15	
Age at RT				0.061
≤64 years	55	38	25	
≥65 years	46	32	16	
Gender				0.30
Female	43	33	30	
Male	56	37	15	
Karnofsky performance score				**<0.001**
≤80	37	24	10	
90–100	73	53	37	
Type of primary tumor				0.26
Breast cancer	50	38	25	
Non-small cell lung cancer	53	38	24	
Small-cell lung cancer	50	28	0	
Less radiosensitive tumors	62	46	23	
Other types	25	17	17	
Number of brain lesions				**0.006**
1–3	58	43	31	
≥4	43	26	9	
Extra-cerebral metastases				0.128
No	55	40	31	
Yes	48	32	14	

RT: Radiotherapy; WBRT: Whole-Brain Radiotherapy; SIB: Simultaneous Integrated Boost. Bold *p*-values are significant.

**Table 3:** Six-month survival rates and related scoring points.

Factor	Survival Rate at 6 Months (%)	Scoring Points
Karnofsky performance score		
90–100	53	5
≤80	24	2
Number of brain lesions		
1–3	43	4
≥4	26	3
Age at radiotherapy		
≤64 years	38	4
≥65 years	32	3
Extra-cerebral metastases		
No	40	4
Yes	32	3

## Data Availability

The data analyzed for this paper cannot be shared due to data protection regulations. According to the ethics committee, only evaluation of anonymized data is allowed for this study.
